# Association of maternal TSH and neonatal metabolism: A large prospective cohort study in China

**DOI:** 10.3389/fendo.2022.1052836

**Published:** 2022-12-01

**Authors:** Qingzhi Hou, Hui Zou, Shuping Zhang, Jiujing Lin, Wenying Nie, Yazhou Cui, Sijin Liu, Jinxiang Han

**Affiliations:** ^1^ Medical Science and Technology Innovation Center, Shandong First Medical University & Shandong Academy of Medical Sciences, Jinan, Shandong, China; ^2^ School of Public Health, Shandong First Medical University & Shandong Academy of Medical Sciences, Jinan, Shandong, China; ^3^ Jinan Maternity and Child Care Hospital Affiliated to Shandong First Medical University, Jinan, Shandong, China; ^4^ Biomedical Sciences College & Shandong Medicinal Biotechnology Centre, Shandong First Medical University & Shandong Academy of Medical Sciences, Jinan, Shandong, China; ^5^ NHC Key Laboratory of Biotechnology Drugs (Shandong Academy of Medical Sciences), Shandong First Medical University & Shandong Academy of Medical Sciences, Jinan, Shandong, China; ^6^ Key Lab for Rare & Uncommon Diseases of Shandong Province, Shandong First Medical University & Shandong Academy of Medical Sciences, Jinan, Shandong, China; ^7^ State Key Laboratory of Environmental Chemistry and Ecotoxicology, Research Center for Eco-Environmental Sciences, Chinese Academy of Sciences, Beijing, China

**Keywords:** pregnancy, maternal TSH, neonatal metabolites, dose-effect relationship, maternal FT4

## Abstract

**Aims:**

Neonatal metabolites are very important in neonatal disease screening, and maternal thyroid hormones play an important role in fetal and neonatal health. Our study aimed to explore the association of maternal thyroid hormones with neonatal metabolites and identify an important time windows.

**Methods:**

Pregnant women were recruited in Jinan Maternity and Child Care Hospital and followed up until delivery. Multivariate generalized linear regression models (GLMs) and restricted cubic spline (RCS) regression analysis models were used to investigate the associations of maternal TSH and FT4 with neonatal metabolites.

**Results:**

In total, 6,653 pairs of mothers and newborns were enrolled in our study. We identified 5 neonatal metabolites, including arginine/ornithine (Arg/Orn), C14:1/C2, C18:1, C3DC+C4OH and C8:1, that were significantly associated with maternal serum TSH during the whole pregnancy (*P* < 0.05), especially in the first trimester. Moreover, 10 neonatal metabolites were significantly associated with maternal serum FT4 (*P* < 0.05), most of which had positive correlations with maternal FT4 in the first trimester (*P* < 0.05). Some neonatal metabolites also had linear or nonlinear dose-effect relationships with maternal serum TSH and FT4 during the whole pregnancy, particularly in the first trimester.

**Conclusions:**

Our study, for the first time, provides epidemiological evidence that maternal serum TSH and FT4, especially during the first trimester, are associated with linear or nonlinear variations in neonatal metabolites. Efforts to identify newborn metabolism levels should carefully consider the effects of maternal thyroid function.

## Introduction

The incidence of neonatal and children’s metabolic and rare diseases is increasing all over the world (1), which could bring great economic and mental burdens to families and society. Newborn blood spot screening (NBS) is a new clinical test for neonatal disease screening (NDS) to detect more than 50 kinds of rare diseases (2, 3). NBS is used to detect the level of circulating blood metabolites within a few hours or days after birth (2, 4, 5), which could be helpful for neonatologists to prevent, diagnose and treat diseases early. Previous studies reported that maternal gestational age, gestational week at delivery (6) and maternal blood glucose levels (7) are associated with neonatal metabolites. The endocrine status of pregnant women, especially thyroid function, plays an important role during pregnancy (8). Thus, exploring maternal thyroid function associated with neonatal metabolites during pregnancy may be an important auxiliary strategy to prevent neonatal diseases.

Thyroid stimulating hormone (TSH) and thyroid hormones (THs), especially FT3 and FT4, which promote amino acid metabolism, protein synthesis (9) and the transfer of fatty acids to mitochondria (10), are required for pregnancy and essential for fetal and newborn development (11–13). For example, maternal TSH and FT4 are required for trophoblast function, early pregnancy maintenance and fetal neurodevelopment (14), and can affect fetal immune function (15) and erythropoiesis (16). The meta-analysis have shown that subclinical maternal hypothyroidism was associated with lower neonatal birth weight, higher maternal FT4 concentrations within the normal range were associated with lower birth weight, and the association between free thyroxine and birth weight was more obvious in the second and third trimesters than in the first trimester (12). Given that the fetal thyroid gland does not produce its own TSH and FT4 in appreciable amounts until the second–third trimester, fetal development largely depends on maternal TSH and FT4 (17–19).

Disordered homeostasis of maternal TSH and THs could increase the frequency of adverse pregnancy outcomes, including intrauterine growth restriction, impaired neurodevelopment and rare diseases (20). As pregnancy progresses, maternal TH levels undergo physiological changes. The fetal thyroid gland begins to develop around the 10th week of pregnancy and becomes functional at approximately midgestation, leading to the requirement of maternal THs in the first trimester of pregnancy (21). During the second and third trimesters of pregnancy and in the first few months after birth, maternal thyroid deficiency can also result in mental and physical retardation and even neurological deficits (9). Deficient maternal TSH and THs in the first trimester can even lead to abortion (22). Neonatal metabolites, especially amino acids and carnitine, play an essential role in fetal growth and development. Inborn errors of metabolism (IEMs) are inherited biochemical disorders that affect physiologically important metabolic pathways (23). Aminoacidopathies is one kind of IEMs caused by abnormal amino acids, and abnormal amino acid structures lead to protein malfunction (24). Carnitine metabolites are associated with infant and newborn growth and development. For example, primary carnitine deficiency can be diagnosed by low free carnitine (C0) levels during neonatal screening (25), and choline deficiency may lead to intrauterine growth restriction and impaired pulmonary and neurocognitive function in newborns (26). Therefore, a large prospective mother-infant cohort study was conducted based on amino acids, carnitine and other metabolites to determine whether neonatal metabolites are affected by maternal serum TSH and FT4 and to explore the dose−response relationship between neonatal metabolites and maternal serum TSH and FT4 during different trimesters.

## Methods

### Cohort enrollment

A mother-infant prospective cohort study was designed to study the correlations of a variety of chemical and nonchemical stressors with maternal health, pregnancy outcomes and child development. Pregnant women who lived in Jinan and had pregnancy tests in the Jinan Maternity and Child Care Hospital from January 2020 to December 2021 (overall range: 5-40 weeks) were enrolled in our mother-infant prospective cohort study and followed up until the delivery of a live singleton infant. In total, 6,653 mother-infant pairs were included.

### Ethics

This study was approved by the Ethics Review Committees of Shandong First Medical University [(R202111290210)] and Jinan Maternity and Child Care Hospital (2022–1–009) and performed in accordance with the Declaration of Helsinki in 1975. Written informed consent was obtained from all participants.

### Measurement of maternal and neonatal thyroid hormones

Maternal serum samples during pregnancy and neonatal heel blood samples within 48 hours after delivery were collected. In detail, 2-3 mL of maternal fasting vein blood was collected in coagulation tubes, centrifuged, and then measured immediately. Maternal serum TSH and FT4 were detected by an electrochemiluminescence microparticle immunoassay system (Architect system, Roche GmbH, Mannheim, Germany) during gestational weeks 5-40. For the neonatal heel blood, 25 μL of blood was applied to Whatman Filter Paper (Sigma Aldrich, USA), followed by neonatal TSH measurement using blood spot standards from an Auto DELFIA kit (DX800, Beckman, USA). Quality control for the TSH test included one duplicate sample measured for every 20 samples to ensure the stability of the whole measurement process, and the sensitivity of the TSH assay was 0.005 mIU/L (27).

### Measurement of neonatal metabolites

In neonatal blood samples, 82 metabolites, including 12 kinds of amino acids and 70 kinds of carnitines, were identified and quantified with nonderivative methods using ultrahigh-performance liquid chromatography-tandem mass spectrometry (UPLC−MS/MS) (Thermo Scientific, Germany). In detail, 3.5-4.0 mm punches were punched from dried blood spots and extracted with 100 μL of 80% aqueous methanol and 0.1% formic acid (Sigma Aldrich, USA) in a thermomixer at 45°C and 700 rpm for 45 min. Extracted liquid was transferred to HPLC vials for direct detection. An ALC system consisting of a Dionex Ultimate 3000 UHPLC quaternary system pump, a column department, and an autosampler (Thermo Scientific, Germany) and a Q Exactive Orbitrap (Thermo Scientific, Germany) were used to analyze the metabolites one by one to obtain molecular weight, structure and other information. Blood samples were tested in both positive and negative modes. The test for the quality control sample was repeatedly conducted every 20 samples to ensure the stability of the whole measurement process. The concentrations of neonatal metabolites were obtained by calculating the signal strength ratio between each analyte and an internal standard.

### Statistical analysis

Continuous variables, which are presented as the means ± SDs, were calculated by the chi-square test when normally distributed. Medians (25th, 75th) are used to express continuous variables with a no normal distribution and were calculated by the Wilcoxon rank sum test, and categorical variables are expressed by the frequency (percentage). Maternal TSH and FT4 levels among different trimesters were compared by Wilcoxon rank sum test. Multivariate Generalized Linear Regression Model (GLM) was used to explore the associations between and maternal TSH and FT4 or neonatal TSH levels and neonatal metabolites with or without adjustment. In GLM models, neonatal metabolite concentrations were standardized to z scores (28) due to the skewed distributions. To further explore the factors affecting maternal TSH and FT4 and neonatal metabolites, subgroup analysis was performed according to different trimesters of maternal serum sample collection and infant sex. Restricted Cubic Splines function (RSC) model (29) was used to explore the dose-effect relationships of maternal or neonatal serum TSH and FT4 with neonatal metabolites with or without adjustment. There were three knots in RCS analysis, including 25^th^, 50^th^ and 75^th^. Covariates for model adjustment in GLM and RSC analyses included the age of pregnant women, gestational weeks of maternal serum sample collection (30), gestational age at delivery, newborn birth weight and infant gender (7), To identify the time-window and dose-effect relationships, subgroup analysis was performed according to different trimesters of maternal serum sample collection and infant gender. All statistical analyses were performed by IBM SPSS version 25.0 and R version 4.1.0. Statistical tests were 2-sided, and a *P* value < 0.05 was considered statistically significant.

## Results

### Baseline characteristics of all subjects and maternal TH levels during pregnancy

In total, 6,653 pregnant women (aged 31.97 ± 4.45) were included for the examination of maternal TSH and FT4 at 5-40 weeks of gestation (13.17 ± 8.07 weeks) (**Table 1**). In detail, TSH and FT4 tests were performed for 4,297 pregnant women in the first trimester (≤ 12 weeks), 1,758 in the second trimester (13-28 weeks), and 598 in the third trimester (29-40 weeks). The newborns (52.6% boys and 47.4% girls) were delivered at an average gestational age of 39.64 ± 1.49 weeks with an average birth weight of 3345.16 ± 51.90 g (**Table 1**). Moreover, the details of the concentrations of neonatal metabolites are shown in **Table S1**.

**Table 1:** Baseline characteristics of all subjects and the concentrations of maternal and neonatal TSH and FT4 and metabolites.

Variables	Value (mean ± SD)	
Age of pregnant women (year)	31.97 ± 4.45
Gestational weeks	13.17 ± 8.07
Gestational age at delivery (week)	39.64 ± 1.49
Birth weight (g)	3345.53 ± 51.90
Gender of newborn, n (%)	
Boy	3499 (52.6%)
Girl	3154 (47.4%)
Maternal serum TSH (mU/L)	1.63 ± 1.27
Maternal serum FT4 (pmol/L)	10.69 ± 2.23
Neonatal TSH (mU/mL)	3.30 ± 2.26

The average concentrations of maternal TSH and FT4 were 1.63 ± 1.27 mU/mL and 10.69 ± 2.23 pmol/L, respectively (**Table 1**). Compared to that from the second to the third trimester, maternal TSH levels in the first trimester were lower (1.52 ± 1.18 vs. 1.84 ± 1.40 mU/mL, *P* < 0.001) (**Table 2**). However, maternal FT4 was higher in the first trimester than in the second and third trimesters (11.47 ± 2.03 vs. 9.27 ± 1.86 pmol/L, *P* < 0.001) (**Table 2**). In addition, the average concentration of neonatal TSH was 3.30 ± 2.26 mU/mL (**Table 1**).

**Table 2:** The concentrations of maternal serum TSH and FT4 during different trimesters.

	Overall	First trimester	Second and third trimesters	
Variables	(n = 6,653)	(n = 4,297)	(n = 2,356)	*P*
TSH (uIU/mL)	1.63 ± 1.27	1.52 ± 1.18	1.84 ± 1.40	< 0.001
FT4 (pmol/l)	10.69 ± 2.23	11.47 ± 2.03	9.27 ± 1.86	< 0.001

### The associations of maternal TSH and FT4 with neonatal metabolites

**Figure 1:**
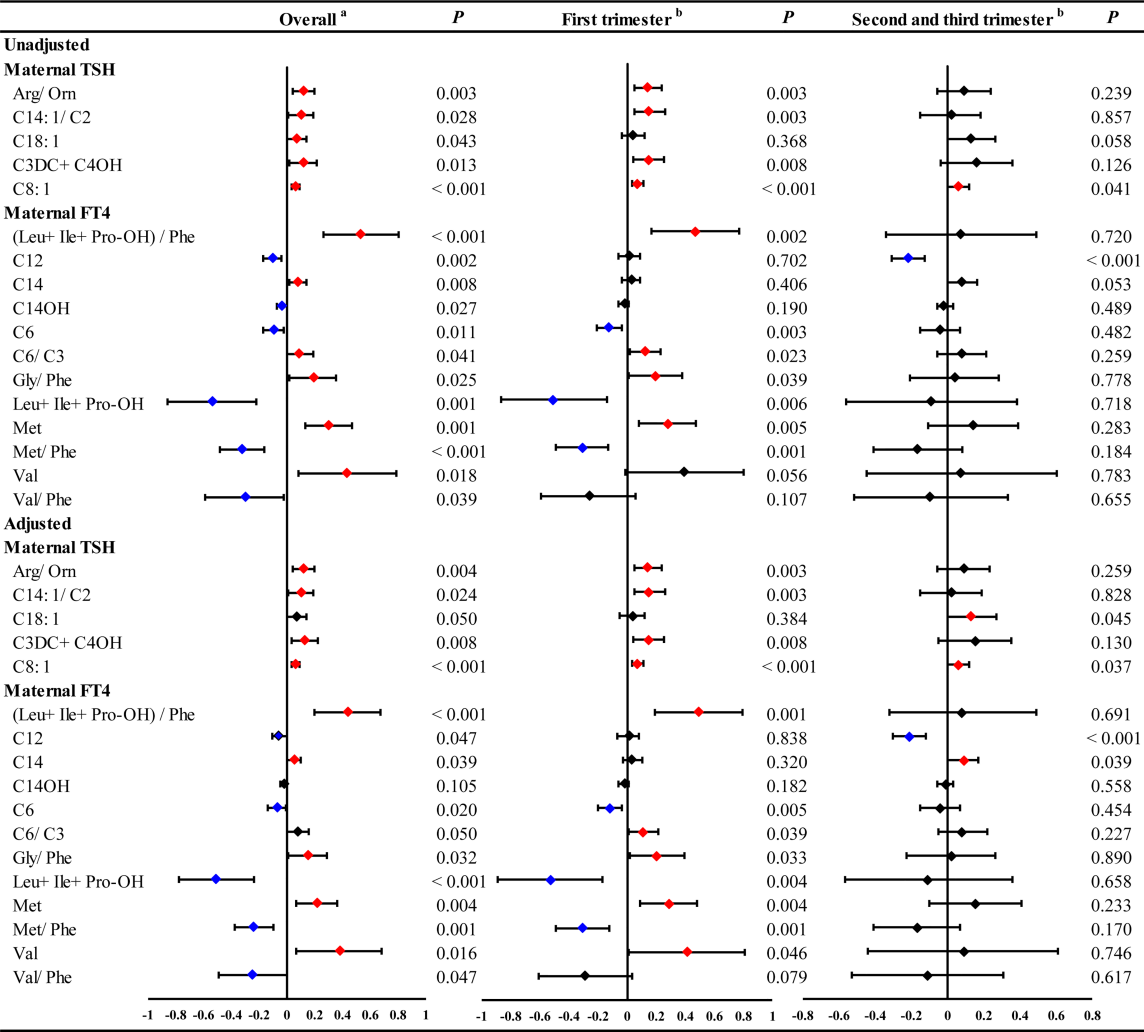
The significant linear ausociations of maternal serum THs with part of neonatal metabolites during overall pregnancy or at different trimesters. GLM Model ^a^adjusted by the age of pregnant women, gestational weeks at sample collection, newborn birth weight and gestational age at delivery. GLM Model ^b^adjusted by the age of pregnant women, newborn birth weight and gestational age at delivery. Red and blue dots indicate significant positive and negative correlations, respectively.

**Figure 2:**
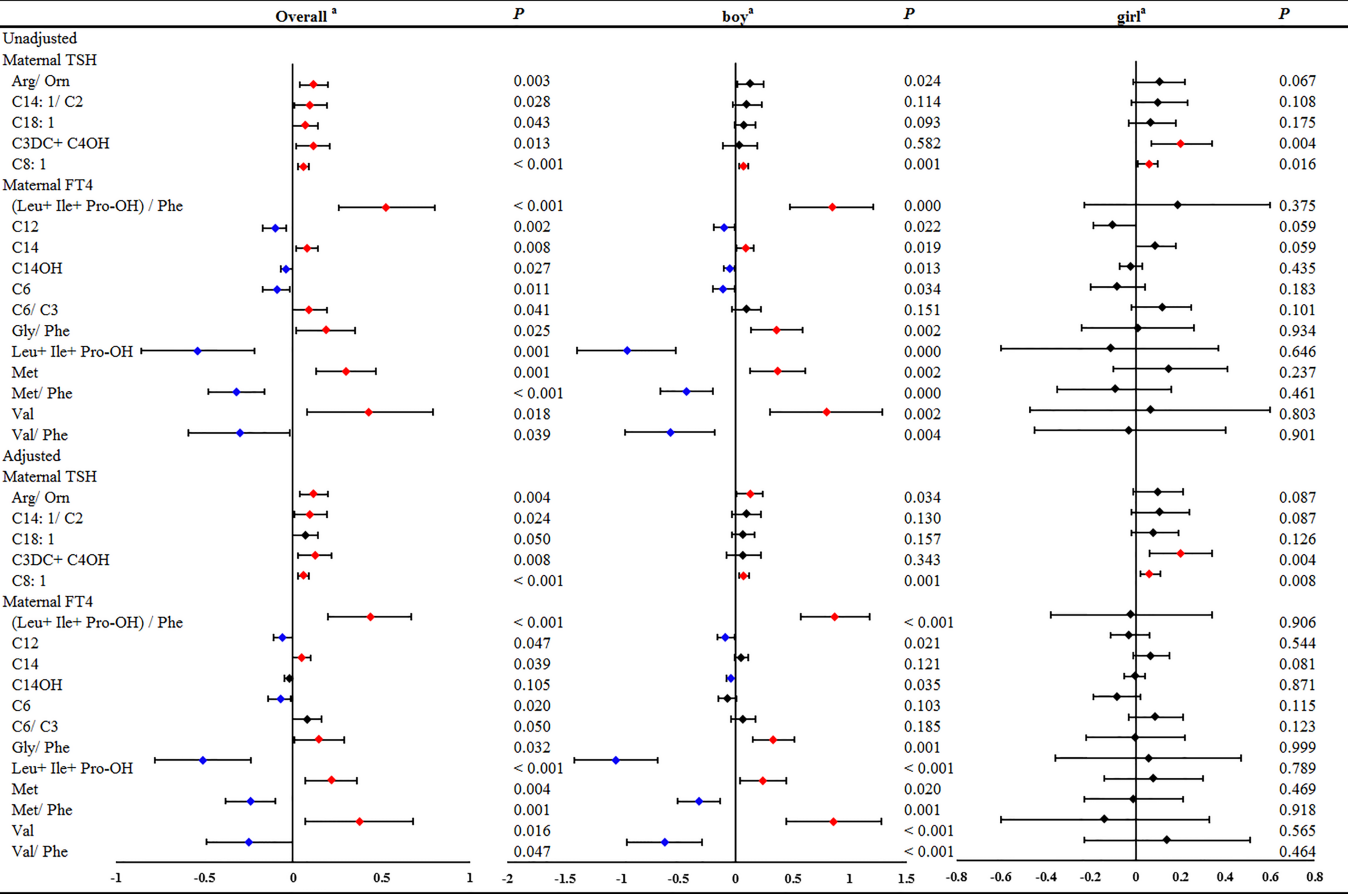
The significant linear associations of maternal serum THs with part of neonatal metabolites during overall pregnancy or in different genders of newborn. GLM Model a: adjusted by the age of pregnant women, gestational weeks at sample collection, newborn birth weight and gestational age at delivery.

In the GLMs model without adjustment for confounding factors, maternal serum TSH was found to be positively associated with 5 neonatal metabolites, including Arg/Orn, C14:1/C2, C18:1, C3DC+C4OH and C8:1 (**Figures 1** and **S1**). However, the stratified analysis by gestational weeks of serum collection showed positive correlations of maternal serum TSH with Arg/Orn, C14:1/C2 and C3DC+C4OH only in the first trimester (**Figure 1**). Consistent results were obtained using GLMs adjusted for maternal age, gestational weeks at maternal serum collection, neonatal birth weight and gestational weeks at delivery (**Figures 1** and **S2**). Maternal serum FT4 was positively associated with neonatal (Leu+Ile+Pro-OH)/Phe, C14, C6/C3, Gly/Phe, Met and Val, but negatively associated with neonatal C12, C14OH, C6, Leu+Ile+Pro-OH, Met/Phe and Val/Phe (**Figures 1** and **S3**). Significant correlations of maternal serum FT4 with these neonatal metabolites were also mostly observed in the first trimester (**Figure 1**). The adjusted GLMs model demonstrated consistent results for most metabolites except C14 and C12, which were only positively and negatively associated with FT4, respectively, during the second and the third trimester (**Figures 1** and **S4**). We also conducted a subgroup analysis according to newborn sex, and the adjusted factors included maternal age, gestational weeks at maternal serum collection, neonatal birth weight and gestational weeks at delivery. The results showed that maternal TSH was positively associated with neonatal Arg/Orn, and maternal serum FT4 was positively associated with neonatal (Leu+Ile+Pro-OH)/Phe, Gly/Phe, Met and Val, but negatively associated with neonatal C12, C14OH, Leu+Ile+Pro-OH, Met/Phe and Val/Phe only in the boy group. Moreover, maternal TSH was positively associated with C3DC+C4OH only in the girl group. Moreover, maternal TSH was positively correlated with C8:1 in both groups (**Figures 2**, **S7–S10**).

Collectively, these results revealed that maternal TSH and FT4 were associated with neonatal metabolites, especially in the first trimester.

### Does-effect relationships of maternal TSH and FT4 with neonatal metabolites

**Figure 3:**
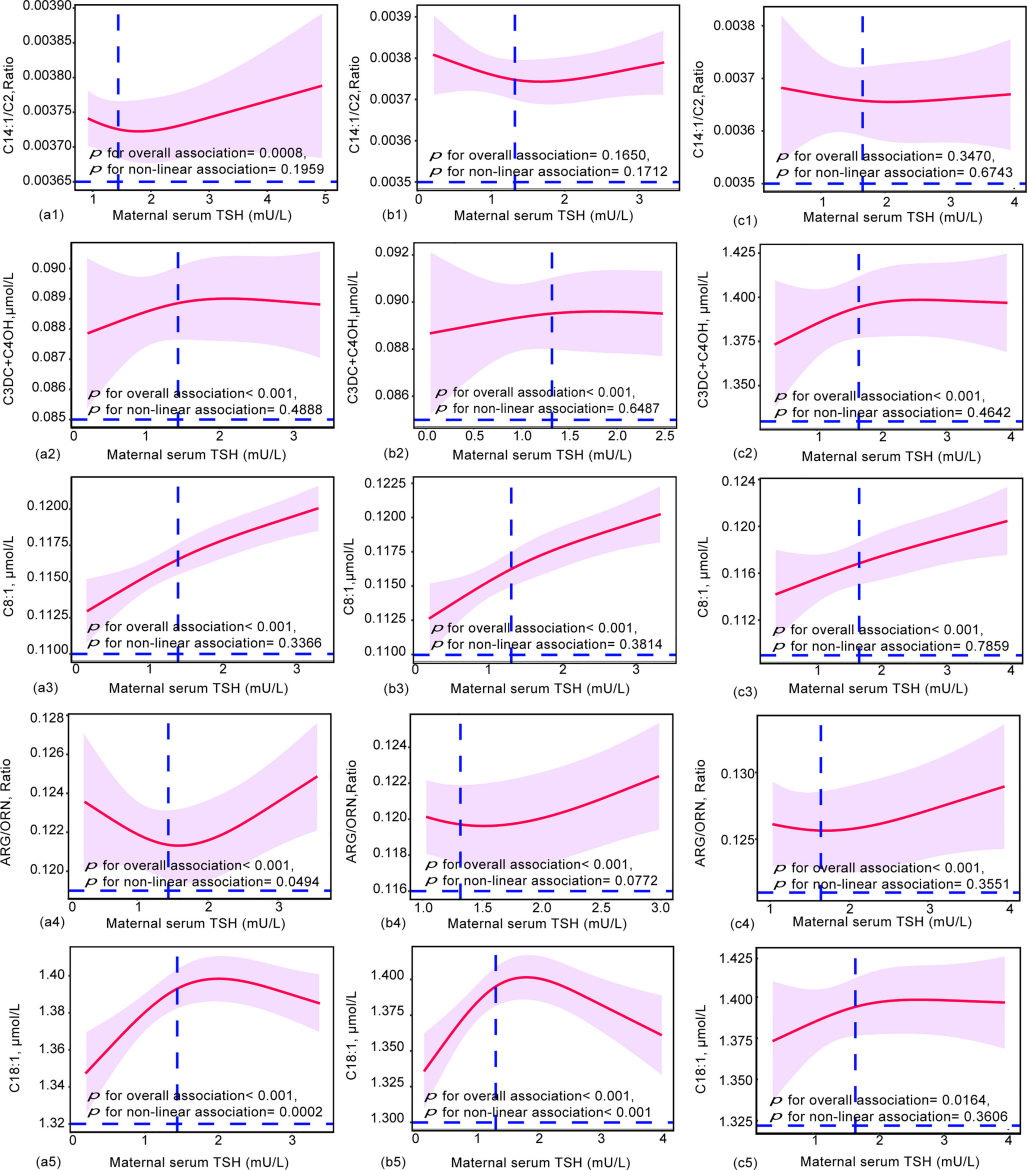
Restricted cubic spline (RCS) regression analysis for the maternal TSH and neonatal metabolites during (a) ( 1-5) overall pregnancy, (b) ( 1-5) at the first trimester, or (c) (1-5) at the second and third trimesters. Neonatal metabolites included were selected by GLM models. The median concentrations of maternal TSH were 1.44 mU/L during overall pregnancy, 1.32 mU/L at the first trimester and 1.65 mU/L at the second and third trimesters. RCS analysis was performed with adjustment by the age of pregnant women, gestational weeks, newborn birth weight and gestational age at delivery.

**Figure 4:**
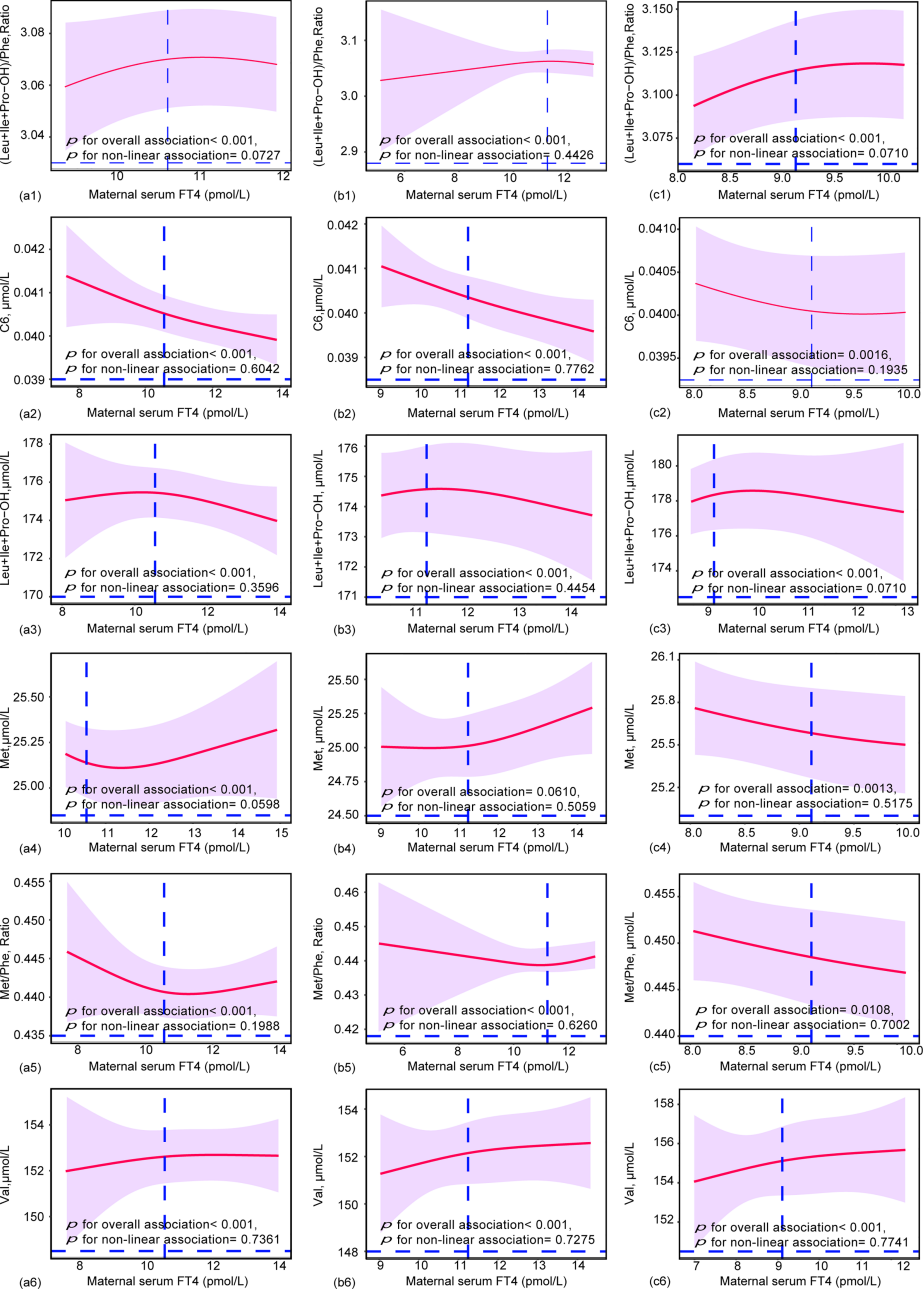
Restricted cubic spline (RCS) regression analysis for the maternal FT4 and neonatal metabolites during (a) (1-5) overall pregnancy, (b) (1-5) at the first trimester, or (c) (1-5) at the second and third trimesters. Neonatal metabolites included were selected by GLM models. The median concentrations of maternal TSH were 10.56 pmol/L during overall pregnancy, 10.25 pmol/L at the first trimester and 9.11 pmol/L at the second and third trimesters. RCS analysis was performed with adjustment by the age of pregnant women, gestational weeks.

To further elucidate the dose-effect relationship between maternal TSH and FT4 and neonatal metabolites, we performed restricted cubic spline (RCS) regression analysis. The results showed that maternal serum TSH had positive linear dose-effect relationships with C8:1 and C3DC+C4OH during the whole pregnancy and in different trimesters (*P* < 0.001 for overall association, *P* > 0.05 for nonlinear association) (**Figure 3**). In detail, we observed a U-shaped dose-effect relationship during the whole pregnancy (*P* < 0.001 for overall association, *P* < 0.001 for nonlinear association) and positive linear dose-effect relationships for each trimester (*P* < 0.01 for overall association, *P* > 0.05 for nonlinear association) between maternal serum TSH and neonatal Arg/Orn. Maternal serum TSH had an inverted U-shaped dose-dependent relationship with C18:1 for the whole pregnancy and the first trimester (*P* < 0.001 for overall association, *P* < 0.001 for nonlinear association) but a positive linear dose-dependent relationship with C18:1 from the second to the third trimesters (*P* = 0.0164 for overall association, *P* = 0.3606 for nonlinear association) (**Figure 3**). Maternal serum FT4 exhibited negative linear dose-effect relationships with C6, Leu+Ile+Pro-OH and Met/Phe and positive linear dose-effect relationships with (Leu+Ile+Pro-OH)/Phe and Val (*P* < 0.001 for overall associations, *P* > 0.05 for nonlinear association) in the whole pregnancy and in different trimesters (**Figure 4**). Maternal serum FT4 had a positive linear dose-effect relationship with Met for the whole pregnancy (*P* for overall associations < 0.001, *P* = 0.0598 for nonlinear association) (**Figure 4**).

Collectively, our results revealed that 5 neonatal metabolites had dose-effect relationships with maternal TSH, while 6 neonatal metabolites had dose-effect relationships with maternal FT4. These relationships were linear or nonlinear.

### The involvement of neonatal TSH in mediating the impacts of maternal TSH and FT4 on neonatal metabolites

To explore whether neonatal TSH was involved in the effects of maternal TSH and FT4 on neonatal metabolites, we further investigated the associations of maternal serum TSH and FT4 with neonatal TSH or metabolites. Maternal TSH was positively associated with neonatal TSH during the whole pregnancy. Maternal serum FT4 only had a positive association with neonatal TSH in the second and third trimesters (**Table S2**). After adjusting for confounding factors, neonatal TSH was positively associated with neonatal (C16+C18:1)/C2, (Leu+Ile+Pro-OH)/Ala, Arg/Orn, Arg/Phe, C14:1/C16, C6, C6DC, C8:1, Cit/Phe and Pro but negatively associated with neonatal (C3DC+C4OH)/C4, (C4DC+C5OH)/C8, Arg, C0/(C16+C18), C14:1, C14:1/C2, C18:1, C18:1OH, C3DC+C4OH, C8/C2, Cit, Orn/Phe and TYR/CIT (**Figure S6**).

**Figure 5:**
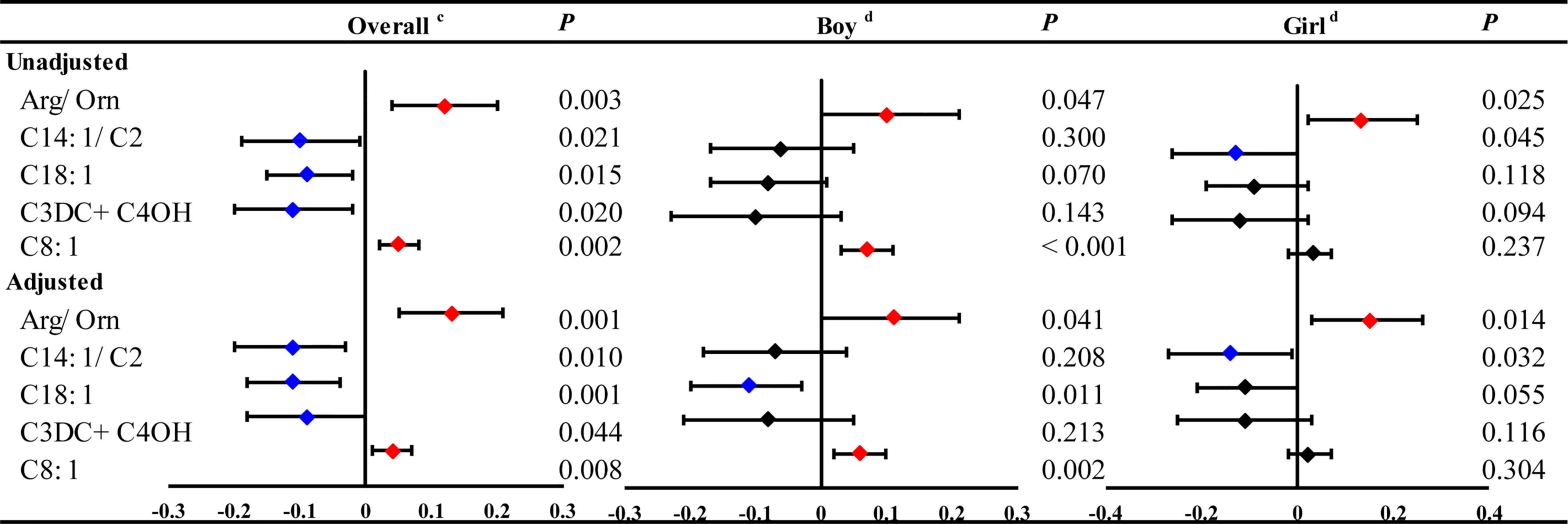
The significant linear associations of maternal and neonatal TSH with neonatal metabolites and stratified by genders in boys or girls. GLM Model ^c^adjusted by the age pregnant women, newborn birth weight, gestational age at delivery and gender of newborns. GLM Model ^d^adjusted by the age of pregnant women, newborn birth weight and gestational age at delivery.

Thus, 5 neonatal metabolites were associated with both maternal and neonatal serum TSH, including Arg/Orn, C14:1/C2, C18:1, C3DC+C4OH and C8:1 (**Figure 5**). Arg/Orn was positively correlated with neonatal TSH in both boys and girls. C8:1 was positively associated with neonatal TSH only in boys, while C14:1/C2 was negatively associated with neonatal TSH only in girls (**Figures 5** and **S6**). These results were in accordance with those from the unadjusted models (**Figures 5** and **S5**).

Taken together, our results demonstrated that maternal TSH and FT4 were associated with neonatal TSH; the latter was also associated with neonatal metabolites, suggesting that neonatal TSH might be involved in mediating the impact of maternal TSH and FT4 on neonatal metabolites. Furthermore, neonatal TSH also showed positive or negative linear dose-effect relationships with different metabolites (**Figure S11**).

## Discussion

Here, for the first time, we investigated the associations of maternal serum TSH and FT4 with neonatal metabolites by performing a large prospective cohort study. We identified 5 metabolites that were significantly associated with maternal and neonatal TSH and 10 metabolites that were significantly associated with maternal serum FT4. Interestingly, maternal serum TSH and FT4 levels were mostly associated with neonatal metabolite levels in the first trimester, indicating that the first trimester might be the an important time for predicting neonatal metabolism by measuring maternal THs. Importantly, all metabolites selected by the GLMs showed linear or nonlinear dose-effect relationships with maternal serum TSH and FT4.

Thyroid hormones have multiple physiological functions and are essential for maintaining normal pregnancy and fetal growth (13). The values of maternal TSH and FT4 dynamically changed during pregnancy. Our present study showed that maternal TSH levels were higher in the first trimester than in the second and third trimesters, while maternal FT4 showed an inverse change. These trends of concentration changes were within the normal range and consistent with previous reports (31–34). Maternal thyroid dysfunction during pregnancy is associated with adverse fetal and neonatal outcomes, including low birth weight (LBW) and neurodevelopmental disorders (17). Medici, Marco et al. (18) revealed that higher but normal FT4 levels in the first trimester were associated with LBW. Allan, W C et al. (35) found that a maternal TSH level >6 mIU/L was significantly associated with a higher rate of fetal death. In addition, various studies have shown that mothers with subclinical or clinical hypothyroidism are at higher risk for fetal distress (19, 36). TSH is considered one of the most important biomarkers for the identification of thyroid dysfunction, and it is closely related to neurodevelopment (37). Although THs are required throughout gestation, the fetal thyroid gland does not produce its own THs in appreciable amounts until the second–third trimester (38). Thyroid deficiency during the last two trimesters of pregnancy and the first few months post-delivery can also result in mental and physical retardation and sometimes neurological deficits (9). For the present study, we explored the associations of maternal serum TSH and FT4 and neonatal metabolites by different trimesters, and we also found that maternal serum TSH was positively associated with neonatal blood TSH throughout the pregnancy period; maternal serum FT4 was positively associated with neonatal blood TSH only in the second and third trimesters, which was in accordance with theoretical knowledge.

Our findings showed that maternal serum TSH and FT4 were associated with neonatal metabolites, including amino acids and carnitine. Most amino acids are positively linked to protein synthesis and pathways for producing cellular energy and are associated with immune functions, especially arginine/ornithine (39). Abnormal concentrations of arginine/ornithine lead to different kinds of diseases, such as heart disease, stroke and other metabolic diseases (9, 40, 41). Increased levels of Val, Leu and Ile are associated with maple syrup urine disease, which is a rare inherited disorder caused by defects in the branched-chain α-ketoacid dehydrogenase complex, leading to elevated branched-chain amino acids in plasma, α-ketoacids in urine and alloisoleucine generation (42). Interestingly, we also found that the correlation between maternal serum TSH and FT4 levels and neonatal TSH was more evident in the first trimester. This was in accordance with previous studies reporting a strong positive association between maternal TSH and neonatal TSH levels (*P*<0.001) (43), and this association was more pronounced in the first trimester (*P*<0.05) (44). An explanation for this is that the fetal thyroid begins to produce thyroid hormone in the second trimester (45). Maternal TSH plays an important role in fetal development, especially in the first trimester. Thus, exploring biomarkers for neonatal metabolism during pregnancy is very important for the prevention of abnormal neonatal metabolite levels.

We also found that maternal and neonatal TSH showed significant linear or nonlinear correlations with neonatal acylcarnitines, including C14:1/C2, C18:1, C3DC+C4OH, and C8:1. Carnitines assist in the transport of long-chain fatty acid CoA into the mitochondria of muscle cells for heat production (4). Carnitines have immunomodulatory, anti-apoptotic and anti-inflammatory effects (46). In particular, elevated levels of C2 and C3, which are involved in the oxidation of branched-chain fatty acids, could also lead to rare diseases, such as methylmalonic acidemia (47). Moreover, disordered carnitines may be associated with fatty acid metabolic diseases and rare diseases (48). Additionally, this was the first epidemiologic study which found that neonatal TSH was statistically associated with 21 neonatal metabolites and that 5 neonatal metabolites were associated with maternal and neonatal serum TSH. Previous epidemiological studies reported that pregnant women with thyroid dysfunction could have serious maternal, fetal and newborn complications, such as spontaneous abortions and preterm birth (33). Hypothyroidism is the most common thyroid dysfunction in pregnancy and is associated with many adverse effects in offspring, including growth retardation, mental retardation, deafness and failure to undergo sexual development (49). Thyroid hormone levels regulate the postnatal increase in growth hormones (50). Moreover, in animal studies, hypothyroidism in fetuses or neonates delayed muscle and nervous system development (51). Above all, maternal TSH and neonatal TSH were associated with neonatal metabolites. Our present study provides an indication for the prevention of neurological and metabolic diseases in offspring.

## Conclusions

Our study provide evidence for the first time that maternal serum TSH and FT4, especially during first trimester were associated with linearly or non-linearly variation of neonatal metabolites. Maternal serum TSH and FT4 during first trimester maybe a biomarker for neonatal metabolite levels, and may be notably central to key processes involved in pregnancy and infant’s growth and development.

## Data availability statement

The raw data supporting the conclusions of this article will be made available by the authors, without undue reservation.

## Ethics statement

The studies involving human participants were reviewed and approved by the Ethics Review Committees of Shandong First Medical University [(R202111290210)] and Jinan Maternity and Child Care Hospital (2022–1–009). Written informed consent to participate in this study was provided by the participants’ legal guardian/next of kin.

## Author contributions

JH: Project administration, conceptualization, methodology, funding acquisition. SZ: Supervision, conceptualization, methodology, investigation, writing - original draft, writing - review and editing, visualization, funding acquisition. QH: Conceptualization, methodology, formal analysis, investigation, data curation, writing - original draft. HZ: Methodology, resources, data curation, writing. WN: Project administration, methodology, resources, data curation. YC: Methodology, resources, data curation, writing. JL: Methodology, resources, data curation. SL: Project administration, conceptualization, funding acquisition. All authors contributed to the article and approved the submitted version.

## Funding

This work was supported under grants from the National Natural Science Foundation of China (grant numbers: 22076104, 91943301 and 21920102007), the International Collaboration Key Grant from the Chinese Academy of Sciences (grant number: 121311KYSB20190010), the “Taishan Scholars” Program for Young Expert of Shandong Province (grant number: tsqn202103105), the “Outstanding University Driven by Talents” Program and Academic Promotion Program of Shandong First Medical University (grant number: 2019LJ001 and 2020LJ002) and the Natural Science Foundation of Shandong Province (grant numbers: ZR2020MH334).

## Conflict of interest

The authors declare that the research was conducted in the absence of any commercial or financial relationships that could be construed as a potential conflict of interest.

## Publisher’s note

All claims expressed in this article are solely those of the authors and do not necessarily represent those of their affiliated organizations, or those of the publisher, the editors and the reviewers. Any product that may be evaluated in this article, or claim that may be made by its manufacturer, is not guaranteed or endorsed by the publisher.

## Glossary

**Table:**

THs	Thyroid hormones
TSH	Thyroid-stimulating hormone
FT4	Serum-free thyroxine
RCS	Restricted Cubic Spline function
GLM	Generalized Linear Regression Model
SD	Standard Deviation
Ala	Alanine
Arg	Arginine
Cit	Citrulline
Gly	Glycine
Met	Methionine
Orn	Ornithine
Phe	Phenylalanine
Pro	Proline
Sa	Succinylacetone
Tyr	Tyrosine
Val	Valine
Leu+Ile+Pro-OH	Leucine family
C0	Free carnitine
C2	Fricanitin
C3	Propionyl carnitine
C4	Butyryl carnitine
C5	Isovaleyanyl carnitine
C5:1	Isovarenyl carnitine
C6	Caproyl carnitine
C6DC	Adipoyl carnitine
C8	Caprylyl carnitine
C8:1	Octenoyl carnitine
C10	Decanoyl carnitine
C10:1	Decanoyl carnitine
C10:2	Sebacyl carnitine
C12	Lauroyl carnitine
C12:1	Dodecenoyl carnitine
C14	Tetracarbonyl carnitine
C14:1	Tetracarbonyl carnitine
C14:2	Tetradecyl carnitine
C14-OH	3⁃Hydroxy tetradecyl carnitine
C16	Cetyl carnitine
C16:1	Hexade ceroyl carnitine
C16:1-OH	3⁃Hydroxyhexadeceroyl carnitine
C16-OH	3⁃Hydroxy hexadecayl carnitine
C18	Octadecyl carnitine
C18:1	Octadeceroyl carnitine
C18:1- OH	3⁃Hydroxyoctadeceroyl carnitine
C18:2	Octadecenoyl carnitine
C18- OH	3⁃Hydroxy octadecanoyl carnitine
IEMs	Inborn errors of metabolism.
