# Supplementary material for: Association of maternal TSH and neonatal metabolism: A large prospective cohort study in China
Source: Front Endocrinol (Lausanne). 2022 Dec 1;13:1052836. doi: 10.3389/fendo.2022.1052836 (PMC9753981; doi:10.3389/fendo.2022.1052836)
Supplement: Supplementary file 1 [file DataSheet_1.docx]

Supplemental materials


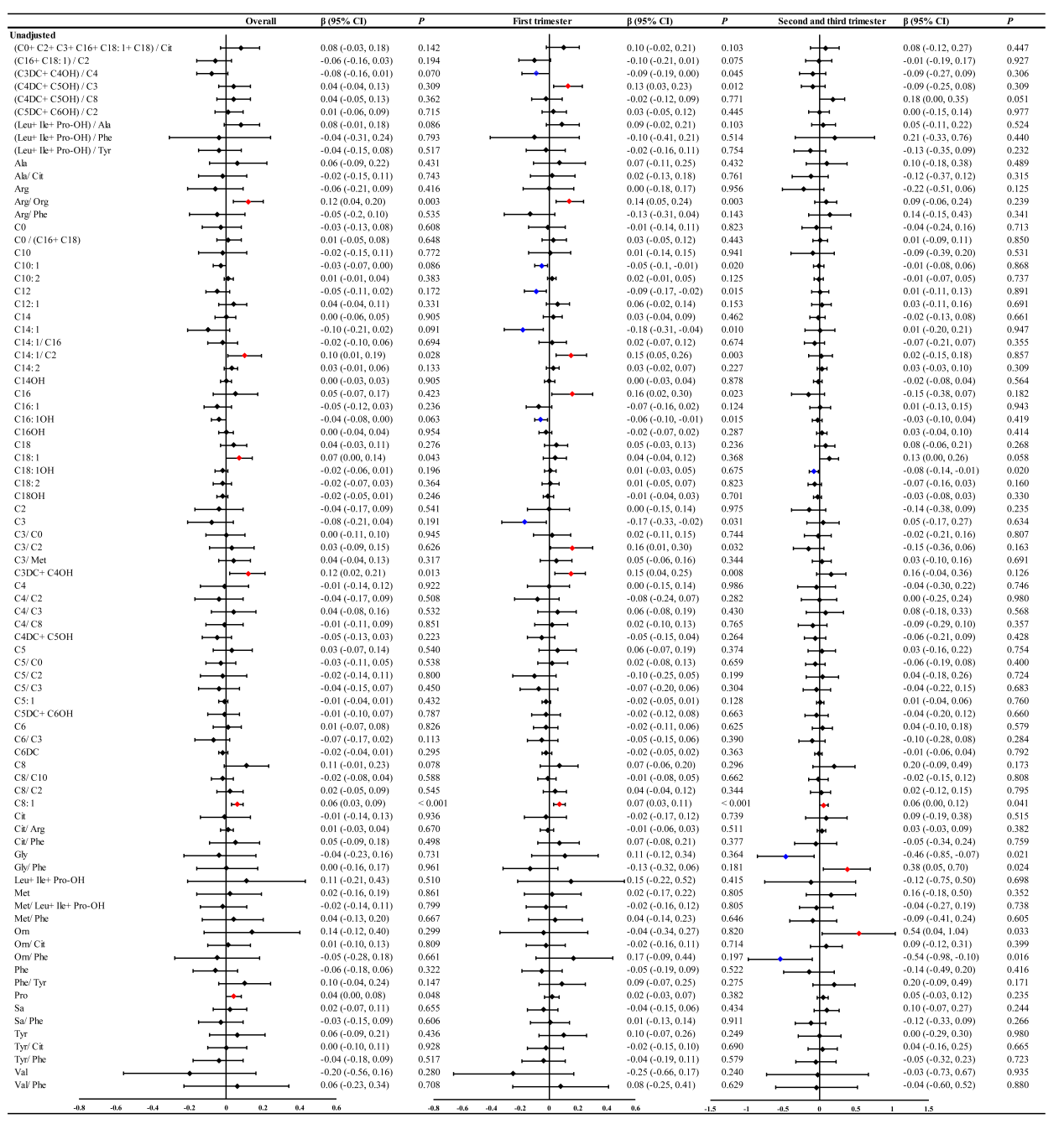


Figure S1. The unadjusted linear associations of maternal TSH and all neonatal metabolites during overall pregnancy or at different trimesters. Red and blue dots indicate significant positive and negative correlations, respectively.


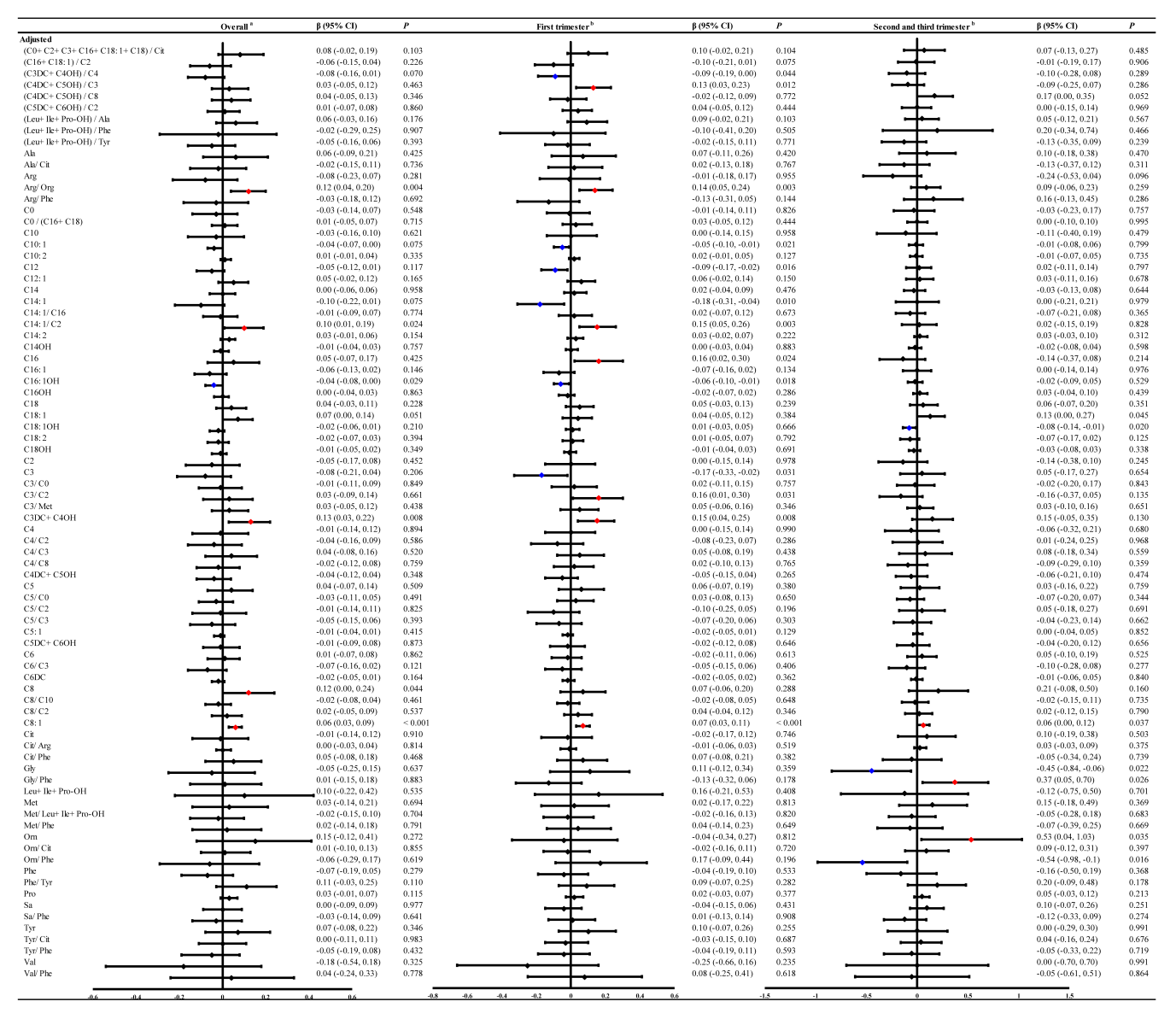


Figure S2. The linear associations of maternal serum TSH with all neonatal metabolites during overall pregnancy or at different trimesters. GLM Model a: adjusted by the age of pregnant women, gestational weeks at sample collection, newborn birth weight and gestational age at delivery. GLM Model b: adjusted by the age of pregnant women, newborn birth weight and gestational age at delivery. Red and blue dots indicate significant positive and negative correlations, respectively.

Figure S3. The unadjusted linear associations of maternal FT4 and all neonatal metabolites during overall pregnancy or at different trimesters. Red and blue dots indicate significant positive and negative correlations, respectively.


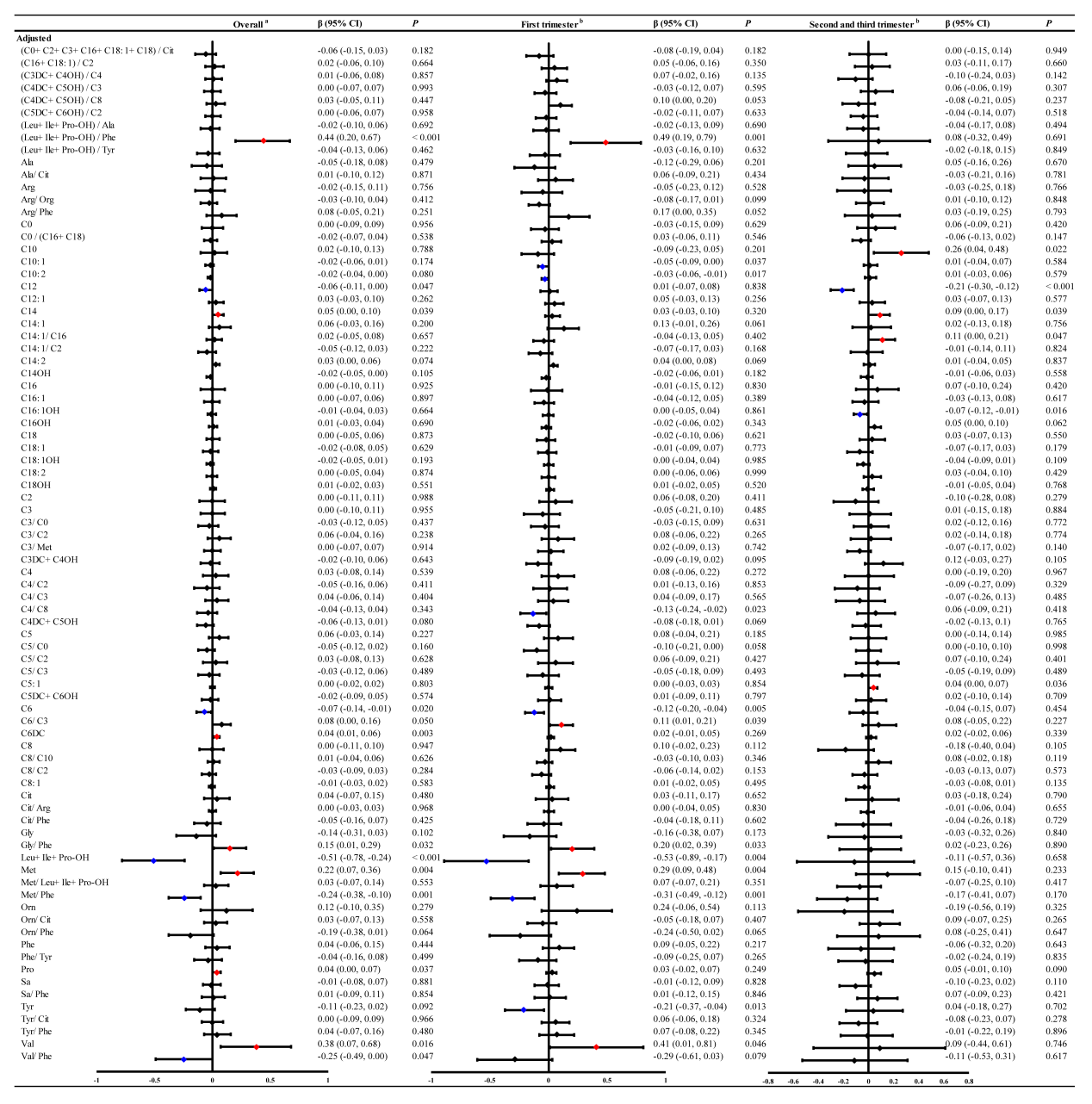


Figure S4. The linear associations of maternal serum FT4 with all neonatal metabolites during overall pregnancy or at different trimesters. GLM Model a: adjusted by the age of pregnant women, gestational weeks at sample collection, newborn birth weight and gestational age at delivery. GLM Model b: adjusted by the age of pregnant women, newborn birth weight and gestational age at delivery. Red and blue dots indicate significant positive and negative correlations, respectively.


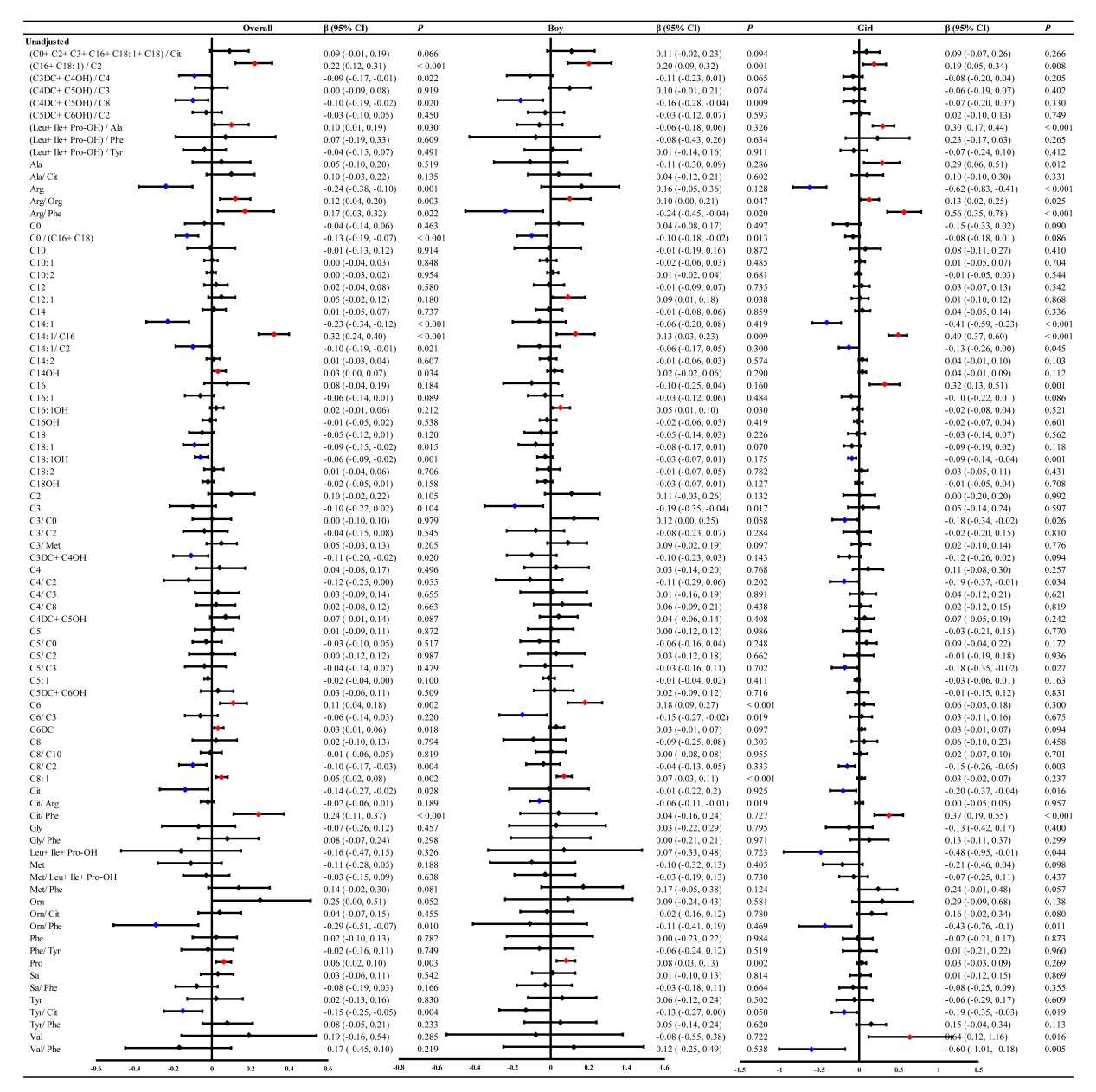


Figure S5. The unadjusted linear associations of neonatal TSH with metabolites for different genders.


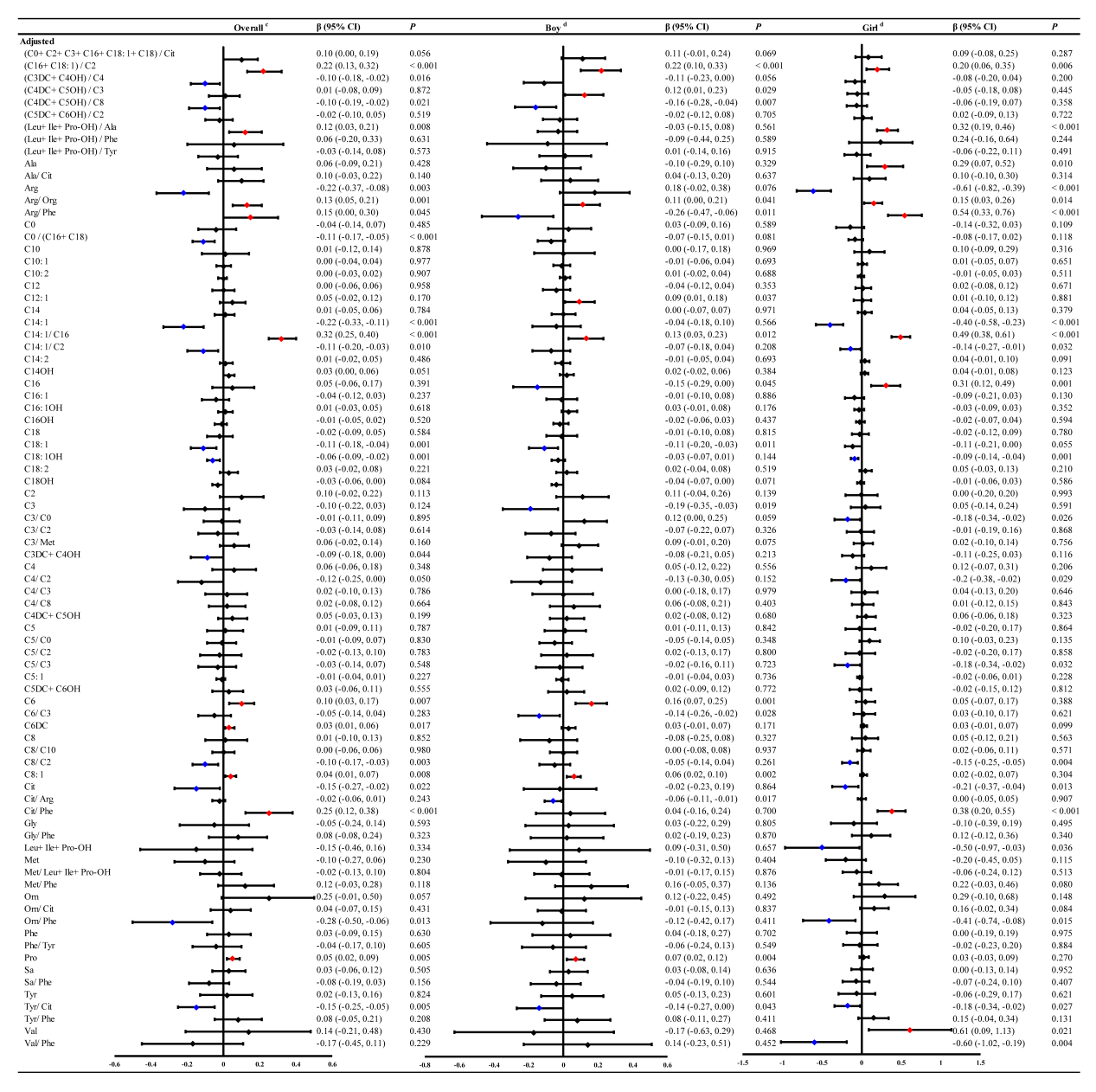


Figure S6. The linear associations of neonatal TSH and metabolites for different genders. Model c: adjusted by the age of pregnant women, newborn birth weight, gestational age at delivery and newborn gender. Model d: adjusted by the age of pregnant women, newborn birth weight and gestational age at delivery.

Figure S7. The unadjusted linear associations of maternal TSH with metabolites for different genders.

Figure S8. The significant linear ausociations of maternal serum THs with part of neonatal metabolites during overall pregnancy or at different gender of newborn. GLM Model a: adjusted by the age of pregnant women, gestational weeks at sample collection, newborn birth weight and gestational age at delivery.

Figure S9. The unadjusted linear associations of maternal FT4 with metabolites for different genders.

Figure S10. The significant linear ausociations of maternal serum FT4 with part of neonatal metabolites during overall pregnancy or at different gender of newborn. GLM Model a: adjusted by the age of pregnant women, gestational weeks at sample collection, newborn birth weight and gestational age at delivery.


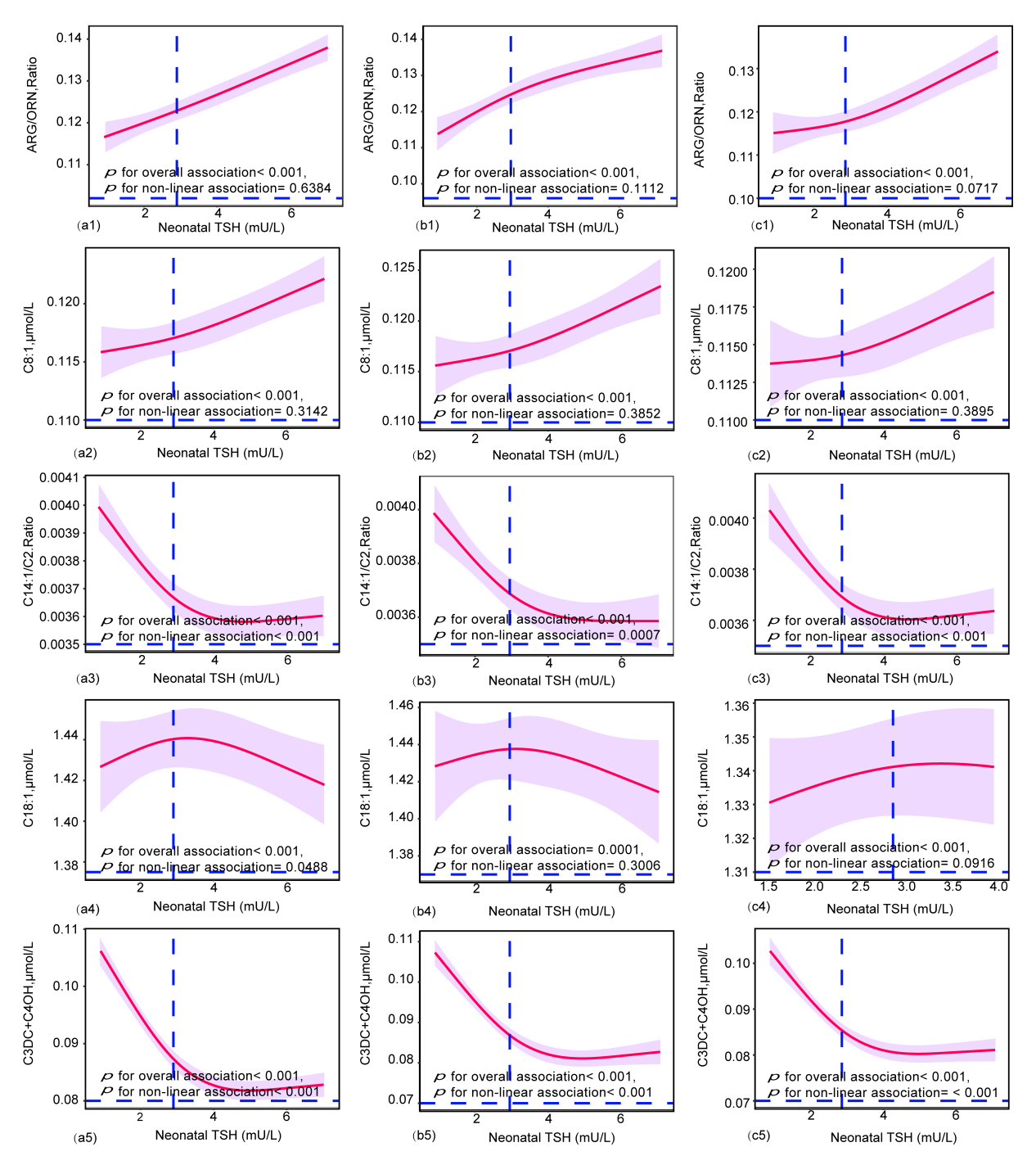


Figure S11. Restricted cubic spline (RCS) regression analysis for the neonatal TSH and neonatal metabolites from both genders (a1-5), boys (b1-5), and girls (c1-5). Neonatal metabolites included were selected by GLM models. The median concentrations of maternal TSH were 2.90 mU/L for both genders, 2.95 mU/L for boys and 2.87 mU/L for girls. RCS analysis was performed with adjustment by the age of pregnant women, gestational weeks, newborn birth weight, newborn gender and gestational age at delivery.

Table S1 The concentrations of neonatal metabolites

| Neonatal metabolites (μmol/ L) | | 25th | 50th | 75th |
| --- | --- | --- | --- | --- |
| **Amino acids** |  |  |  |  |
| Ala |  | 381.59 | 325.27 | 449.17 |
| Arg |  | 15.95 | 10.71 | 22.72 |
| Cit |  | 13.96 | 11.58 | 16.83 |
| Gly |  | 439.23 | 372.12 | 522.76 |
| Orn |  | 134.23 | 108.40 | 169.59 |
| Phe |  | 56.62 | 50.28 | 64.19 |
| Sa |  | 0.66 | 0.56 | 0.73 |
| Tyr |  | 104.40 | 84.26 | 131.58 |
| Pro |  | 187.81 | 163.98 | 217.13 |
| Met |  | 24.71 | 20.81 | 29.10 |
| Val |  | 147.50 | 127.36 | 171.32 |
| Leu+Ile+Pro-OH | | 169.86 | 146.44 | 197.50 |
| **Ratios of different amino acids** | |  |  |  |
| Ala/Cit |  | 27.42 | 22.14 | 33.24 |
| Arg/Phe |  | 0.28 | 0.19 | 0.39 |
| Cit/Arg |  | 0.88 | 0.63 | 1.29 |
| Cit/Phe |  | 0.25 | 0.21 | 0.30 |
| Gly/Phe |  | 7.82 | 6.67 | 9.14 |
| Orn/Cit |  | 9.67 | 7.87 | 11.80 |
| Orn/Phe |  | 2.39 | 1.95 | 2.95 |
| Phe/Tyr |  | 0.55 | 0.44 | 0.67 |
| Sa/Phe |  | 0.01 | 0.01 | 0.13 |
| Tyr/Cit |  | 7.41 | 5.81 | 9.69 |
| Tyr/Phe |  | 1.83 | 1.50 | 2.25 |
| (Leu+Ile+Pro-OH)/Ala | | 0.45 | 0.39 | 0.51 |
| (Leu+Ile+Pro-OH)/Tyr | | 1.64 | 1.35 | 1.98 |
| Met/(Leu+Ile+Pro-OH) | | 0.15 | 0.13 | 0.17 |
| Arg/Orn |  | 0.12 | 0.08 | 0.16 |
| Met/Phe |  | 0.43 | 0.37 | 0.50 |
| Val/Phe |  | 2.62 | 2.29 | 2.96 |
| (Leu+Ile+Pro-OH)/Phe | | 2.99 | 2.63 | 3.40 |
| **Carnitines** |  |  |  |  |
| C0 |  | 22.02 | 18.36 | 26.53 |
| C2 |  | 19.39 | 15.96 | 23.54 |
| C3 |  | 1.82 | 1.43 | 2.34 |
| C4 |  | 0.21 | 0.18 | 0.25 |
| C5 |  | 0.11 | 0.09 | 0.13 |
| C5:1 |  | 0.01 | 0.01 | 0.01 |
| C6 |  | 0.04 | 0.03 | 0.05 |
| C8 |  | 0.04 | 0.04 | 0.06 |
| C8:1 |  | 0.11 | 0.09 | 0.14 |

continuing Table S1 The concentrations of neonatal metabolites

| Neonatal metabolites (μmol/ L) | | 25th | 50th | 75th |
| --- | --- | --- | --- | --- |
| **Carnitines** |  |  |  |  |
| C10 |  | 0.06 | 0.04 | 0.07 |
| C10:1 |  | 0.06 | 0.05 | 0.08 |
| C10:2 |  | 0.01 | 0.01 | 0.01 |
| C12 |  | 0.06 | 0.05 | 0.07 |
| C12:1 |  | 0.04 | 0.03 | 0.05 |
| C14 |  | 0.16 | 0.13 | 0.19 |
| C14:1 |  | 0.07 | 0.06 | 0.08 |
| C14:2 |  | 0.02 | 0.02 | 0.02 |
| C14OH |  | 0.01 | 0.01 | 0.01 |
| C16 |  | 2.70 | 2.13 | 3.36 |
| C16:1 |  | 0.13 | 0.10 | 0.17 |
| C16OH |  | 0.01 | 0.01 | 0.02 |
| C16:1OH |  | 0.04 | 0.03 | 0.05 |
| C18 |  | 0.76 | 0.62 | 0.92 |
| C18:1 |  | 1.34 | 1.12 | 1.60 |
| C18:1OH |  | 0.02 | 0.01 | 0.02 |
| C18:2 |  | 0.29 | 0.23 | 0.36 |
| C18OH |  | 0.01 | 0.01 | 0.01 |
| C3DC+C4OH |  | 0.08 | 0.06 | 0.10 |
| C4DC+C5OH |  | 0.19 | 0.16 | 0.23 |
| C5DC+C6OH |  | 0.10 | 0.08 | 0.12 |
| C6DC |  | 0.08 | 0.06 | 0.10 |
| C0/(C16+C18) | | 6.42 | 5.13 | 8.12 |
| C3/C0 |  | 0.08 | 0.07 | 0.10 |
| C3/C2 |  | 0.09 | 0.08 | 0.12 |
| C3/Met |  | 0.07 | 0.06 | 0.10 |
| C4/C2 |  | 0.01 | 0.01 | 0.01 |
| C4/C3 |  | 0.12 | 0.09 | 0.15 |
| C4/C8 |  | 4.80 | 3.80 | 6.00 |
| C5/C0 |  | 0.01 | 0.00 | 0.01 |
| C5/C2 |  | 0.01 | 0.00 | 0.01 |
| C5/C3 |  | 0.06 | 0.05 | 0.07 |
| C6/C3 |  | 0.02 | 0.02 | 0.03 |
| C8/C2 |  | 0.00 | 0.00 | 0.00 |
| C8/C10 |  | 0.78 | 0.67 | 0.86 |
| C14:1/C2 |  | 0.00 | 0.00 | 0.00 |
| C14:1/C16 |  | 0.03 | 0.02 | 0.03 |
| (C16+C18:1)/C2 | | 0.21 | 0.18 | 0.24 |
| (C3DC+C4OH)/C4 | | 0.38 | 0.30 | 0.47 |
| (C4DC+C5OH)/C3 | | 0.10 | 0.08 | 0.13 |
| (C4DC+C5OH)/C8 | | 4.25 | 3.26 | 5.67 |
| (C5DC+C6OH)/C2 | | 0.00 | 0.01 | 0.01 |
| (C0+C2+C3+C16+C18:1+C18)/Cit | | 3.49 | 2.75 | 4.40 |

Table S2 The linear associations of neonatal serum TSH with maternal serum THs

|  | **Overall ^a^** | ***P*** | **First trimester ^b^** | ***P*** | **Second and third**  **Trimesters ^b^** | | ***P*** |
| --- | --- | --- | --- | --- | --- | --- | --- |
|  | **β (95% CI)** |  | **β (95% CI)** |  | **β (95% CI)** | |  |
| **Unadjusted** | |  |  |  |  |  | |
| TSH | 0.09 (0.06, 0.11) | < 0.001 | 0.09 (0.06, 0.13) | < 0.001 | 0.08 (0.05, 0.12) | < 0.001 | |
| FT4 | 0.01 (-0.02, 0.03) | 0.652 | -0.02 (-0.05, 0.02) | 0.355 | 0.05 (0.00, 0.10) | 0.032 | |
| **Adjusted** |  |  |  |  |  |  | |
| TSH | 0.09 (0.07, 0.12) | < 0.001 | 0.10 (0.06, 0.13) | < 0.001 | 0.09 (0.05, 0.12) | < 0.001 | |
| FT4 | 0.01 (-0.02, 0.03) | 0.059 | -0.02 (-0.05, 0.02) | 0.302 | 0.05 (0.01, 0.11) | <0.001 | |

GLM Model a: adjusted by the age of pregnant women, gestational weeks of serum collected, newborn birth weight and gestational age at delivery. GLM Model b: adjusted by the age of pregnant women, newborn birth weight and gestational age at delivery.

Setting of amino acid, free carnitine, acyl carnitine for neonatal disease screening by LC-MS

|  | | **Name of metabolite** |  | **Non-derivatization [M+H]^+^** |
| --- | --- | --- | --- | --- |
| Ala |  | Alanine |  | 90.1 |
| Arg |  | Arginine |  | 175.1 |
| Cit |  | Citrulline |  | 176.1 |
| Gly |  | Glycine |  | 76.1 |
| Met |  | Methionine |  | 150.1 |
| Orn |  | Ornithine |  | 133.1 |
| Phe |  | Phenylalanine |  | 166.1 |
| Pro |  | Proline |  | 116.1 |
| Sa |  | Succinylacetone |  | - |
| Tyr |  | Tyrosine |  | 182.1 |
| Val |  | Valine |  | 118.1 |
| Leu+Ile+Pro-OH | | Leucine family |  | - |
| C0 |  | Free carnitine |  | 162.1 |
| C2 |  | Fricanitin |  | 204.1 |
| C3 |  | Propionyl carnitine |  | 218.1 |
| C4 |  | Butyryl carnitine |  | 232.2 |
| C5 |  | Isovaleyanyl carnitine |  | 246.2 |
| C5:1 |  | Isovarenyl carnitine |  | 244.2 |
| C6 |  | Caproyl carnitine |  | 260.2 |
| C6DC |  | Adipoyl carnitine |  | 290.2 |
| C8 |  | Caprylyl carnitine |  | 288.2 |
| C8:1 |  | Octenoyl carnitine |  | 286.2 |
| C10 |  | Decanoyl carnitine |  | 316.3 |
| C10:1 |  | Decanoyl carnitine |  | 314.2 |
| C10:2 |  | Sebacyl carnitine |  | 312.2 |
| C12 |  | Lauroyl carnitine |  | 344.3 |
| C12:1 |  | Dodecenoyl carnitine |  | 342.3 |
| C14 |  | Tetracarbonyl carnitine |  | 372.3 |
| C14:1 |  | Tetracarbonyl carnitine |  | 370.3 |
| C14:2 |  | Tetradecyl carnitine |  | 368.3 |
| C14-OH |  | 3⁃Hydroxy tetradecyl carnitine | | 388.3 |
| C16 |  | Cetyl carnitine |  | 400.3 |
| C16:1 |  | Hexadeceroyl carnitine |  | 398.3 |
| C16:1-OH | | 3⁃Hydroxyhexadeceroyl carnitine | | 414.3 |
| C16-OH |  | 3⁃Hydroxy hexadecayl carnitine | | 416.3 |
| C18 |  | Octadecyl carnitine |  | 428.4 |
| C18:1 |  | Octadeceroyl carnitine |  | 426.4 |
| C18:1-OH | | 3⁃Hydroxyoctadeceroyl carnitine | | 442.4 |
| C18:2 |  | Octadecenoyl carnitine |  | 424.3 |
| C18-OH |  | 3⁃Hydroxy octadecanoyl carnitine | | 444.4 |

[M+H]^+^: Protonated molecular ion

Abbreviation of Amino acid ratio index and Acyl carnitine ratio index

| Amino acid ratio index | | Acyl carnitine ratio index |
| --- | --- | --- |
| Ala/Cit |  | C0/(C16+C18) |
| Arg/Phe | | C3/C0 |
| Cit/Arg |  | C3/C2 |
| Cit/Phe |  | C3/Met |
| Gly/Phe | | C4/C2 |
| Orn/Cit |  | C4/C3 |
| Orn/Phe | | C4/C8 |
| Phe/Tyr | | C5/C0 |
| Sa/Phe |  | C5/C2 |
| Tyr/Cit |  | C5/C3 |
| Tyr/Phe | | C6/C3 |
| Arg/Orn | | C8/C2 |
| Met/Phe | | C8/C10 |
| Val/Phe | | C14:1/C2 |
| (Leu+Ile+Pro-OH)/Phe | | C14:1/C16 |
| (Leu+Ile+Pro-OH)/Ala | | (C16+C18:1)/C2 |
| (Leu+Ile+Pro-OH)/Tyr | | (C3DC+C4OH)/C4 |
| Met/(Leu+Ile+Pro-OH) | | (C4DC+C5OH)/C3 |
|  | | (C4DC+C5OH)/C8 |
|  | | (C5DC+C6OH)/C2 |
|  | | (C0+C2+C3+C16+C18:1+C18)/Cit |
